# TbasCO: trait-based comparative ‘omics identifies ecosystem-level and niche-differentiating adaptations of an engineered microbiome

**DOI:** 10.1038/s43705-022-00189-2

**Published:** 2022-11-07

**Authors:** E. A. McDaniel, J. J. M. van Steenbrugge, D. R. Noguera, K. D. McMahon, J. M. Raaijmakers, M. H. Medema, B. O. Oyserman

**Affiliations:** 1 Department of Bacteriology, University of Wisconsin—Madison, Madison, WI USA; 2 Microbiology Doctoral Training Program, University of Wisconsin—Madison, Madison, WI USA; 3 Bioinformatics Group, Wageningen University and Research, Wageningen, The Netherlands; 4 Microbial Ecology, Netherlands Institute of Ecological Research, Wageningen, The Netherlands; 5 Laboratory of Nematology, Wageningen University, Wageningen, The Netherlands; 6 Department of Civil and Environmental Engineering, University of Wisconsin—Madison, Madison, WI USA; 7 Institute of Biology, Leiden University, Leiden, Netherlands

**Keywords:** Metagenomics, Applied microbiology

## Abstract

A grand challenge in microbial ecology is disentangling the traits of individual populations within complex communities. Various cultivation-independent approaches have been used to infer traits based on the presence of marker genes. However, marker genes are not linked to traits with complete fidelity, nor do they capture important attributes, such as the timing of gene expression or coordination among traits. To address this, we present an approach for assessing the trait landscape of microbial communities by statistically defining a trait attribute as a shared transcriptional pattern across multiple organisms. Leveraging the KEGG pathway database as a trait library and the Enhanced Biological Phosphorus Removal (EBPR) model microbial ecosystem, we demonstrate that a majority (65%) of traits present in 10 or more genomes have niche-differentiating expression attributes. For example, while many genomes containing high-affinity phosphorus transporter *pstABCS* display a canonical attribute (e.g. up-regulation under phosphorus starvation), we identified another attribute shared by many genomes where transcription was highest under high phosphorus conditions. Taken together, we provide a novel framework for unravelling the functional dynamics of uncultivated microorganisms by assigning trait-attributes through genome-resolved time-series metatranscriptomics.

## Introduction

A longstanding cornerstone of deterministic ecological theory is that the environment selects for traits. Traits may be defined as any physiological, morphological, or genomic signature that affects the fitness or function of an individual [1]. Trait-based approaches have become indispensable in macroecological systems to describe fitness trade-offs and the effects of biodiversity on ecosystem functioning [2–5]. Recently, trait-based frameworks have been proposed as an alternative to taxonomy-based methods for describing microbial ecosystem processes [6, 7]. Connecting microbial traits and their phylogenetic distributions to ecosystem-level functions can provide powerful insights into the ecological and evolutionary dynamics underpinning community assembly, microbial biogeography, and organismal responses to changes in the environment [8–10]. Additionally, pinpointing the organismal distribution of traits and the ecological selective pressures that enrich them may be leveraged to reproducibly and rationally engineer stable, functionally redundant ecosystems [11–15]. However, applying trait-based approaches to microbial communities is challenging due to the difficulty in identifying and measuring relevant ecological traits for a given ecosystem [16].

**Fig. 1:**
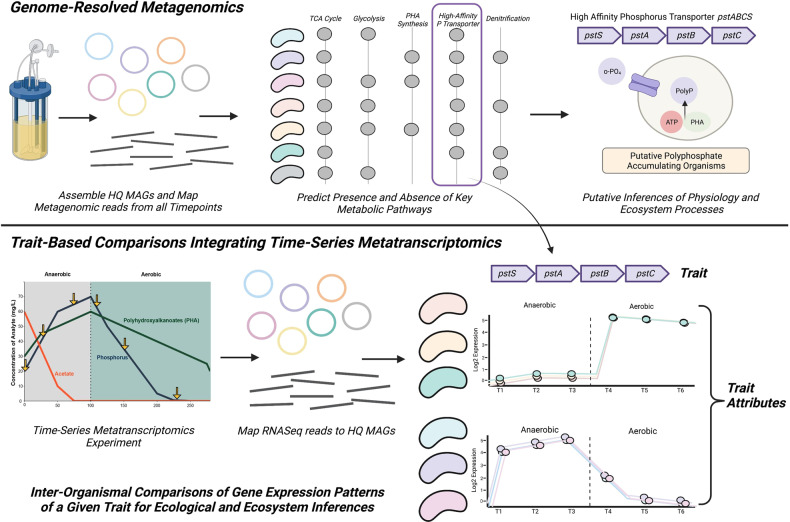
Overview of trait-based comparative transcriptomics approach In genome-resolved metagenomics approaches, representative MAGs are assembled from a microbial community of interest, and the presence and/or absence of key metabolic pathways are used to make inferences of metabolic potential and ecosystem processes. However, metagenomic data alone can only assess the metabolic potential of a given pathway, and do not provide other biologically relevant information such as the timing or induction of these traits. Using time-series metatranscriptomics, we developed a trait-based comparative ‘omics (TbasCO) pipeline that statistically assesses the inter-organismal differences in gene expression pattern of a given trait to cluster into trait attributes. As expression patterns are determined by the time-points assessed in an experiment, it is important to design the sampling regime to capture relevant ecophysiological changes within the ecosystem.

High-throughput sequencing technologies and multi-omics techniques are now routinely used to describe the diversity, activity, and functional potential of uncultivated microbial lineages [17–21]. Improvements in bioinformatics algorithms, and in particular metagenomic binning methods, have allowed for genome-resolved investigations of microbial communities rather than gene-based analyses of assembled contigs [22]. These (meta) genomes are subsequently leveraged to detect the presence of key genes or pathways and predict specific traits of the whole community [19, 23]. Integrating metatranscriptomics data addresses a key limitation, as expression patterns better reflect the actual functional dynamics of a trait compared to gene presence alone. Here, we present TbasCO, a software package and statistical framework for *T*rait-*bas*ed *C*omparative ‘*O*mics to identify expression attributes. We adopt the terminology *attribute* as a hierarchically structured feature of a trait and assert that statistically similar transcriptional patterns of traits across multiple organisms be treated as *attributes* (Fig. 1). This new terminology addresses two key semantic challenges. First, by extending upon the current usage of the term “trait” for the presence and absence of pathways to the corresponding transcriptional patterns. Second, it addresses a limitation of the terminology of “co-expression”, which becomes biologically inaccurate when comparing across independent populations of organisms within a community. In this manner, the identification of expression-based *attributes* provides a high-throughput and intuitive framework for extending trait-based methods to time-series expression patterns in microbial communities. We implement this trait-based approach to classify transcriptional attributes in a microbial community performing Enhanced Biological Phosphorus Removal (EBPR), a globally important biotechnological process implemented in numerous wastewater treatment plants (WWTPs).

The fundamental feature of the engineered EBPR ecosystem is the decoupled and cyclic availability of an external carbon source and terminal electron acceptor. This cycling is often referred to as “feast-famine” conditions and provides a strong selective pressure for traits such as polymer cycling. Accumulation of intracellular polyphosphate through cyclic anaerobic-aerobic conditions ultimately results in net phosphorus removal and accomplishes the EBPR process [24, 25]. One of the most well-studied polyphosphate accumulating organisms (PAOs) belongs to the uncultivated bacterial lineage ‘*Candidatus* Accumulibacter phosphatis’ (hereby referred to as Accumulibacter) [24, 26]. Numerous genome-resolved ‘omics methods have been used to investigate the physiology and regulation of this model PAO enriched in engineered lab-scale enrichment bioreactor systems [27–35]. However, novel and putative PAOs have been discovered that remove phosphorus without exhibiting the hallmark traits of Accumulibacter [36–41]. Additionally, although these lab-scale systems are designed to specifically enrich for Accumulibacter, a diverse bacterial community persists in these environments [27], and their ecological roles have largely remained unexplored. As a result, the general adaptations of microbial lineages inhabiting the EBPR community are not well understood. Using genome-resolved metagenomics and metatranscriptomics, we assembled 66 species-representative genomes spanning several significant EBPR lineages and identified the distribution of expression-based attributes. We show that while some expression attributes are distributed in few genomes, many are redundant and shared across many lineages. Furthermore, we find that a majority of core traits (as defined by the presence of marker genes) have multiple attributes, suggesting that identifying niche-differentiating expression attributes may be used to reveal a large hidden metabolic versatility when investigating genomic data alone.

## Materials and methods

### Metagenomic assembly, annotation, and metatranscriptomic mapping

Three metagenomes sampled from an EBPR bioreactor in May of 2013 with linked time-series metatranscriptomics data were sequenced [42]. Samples were collected and DNA extracted according to the Supplemental Methods. Metagenomic samples were processed and assembled into 66 species-representative bins as described in detail in the Supplemental Methods. All bins are greater than 75% complete and contain less than 10% contamination, with a large majority (44/66) >95% complete and <5% redundant as calculated by CheckM [43] and are all described in Table 1.

**Table 1:** Genome quality statistics and relative abundance calculations for all 66 EBPR SBR MAGs.

Code	Genbank accession	Classification	Completeness	Contamination	Size (Mbp)	Contigs	GC	Abundance 2013-5-13	Abundance 2013-5-23	Abundance 2013-5-28	Total Transcriptional Reads Mapped	Total rRNAs	Total tRNAs
AUS1	GCA_020161845.1	d__Bacteria;p__Actinobacteriota;c__Actinobacteria;o__Actinomycetales;f__Dermatophilaceae;g__Austwickia;s__	99.45	5.01	4.39	82	71.2	0.261	0.720	0.124	255331	3	61
PHYC1	GCA_020161815.1	d__Bacteria;p__Actinobacteriota;c__Actinobacteria;o__Actinomycetales;f__Dermatophilaceae;g__Phycicoccus;s__	98.02	0.54	3.06	34	71	1.355	3.007	0.341	332509	1	49
PHYC2	GCA_020161155.1	d__Bacteria;p__Actinobacteriota;c__Actinobacteria;o__Actinomycetales;f__Dermatophilaceae;g__Phycicoccus;s__Phycicoccus	95.82	1.89	3.20	111	69.2	0.047	0.174	0.112	152031	1	52
TET1	GCA_020160805.1	d__Bacteria;p__Actinobacteriota;c__Actinobacteria;o__Actinomycetales;f__Dermatophilaceae;g__Tetrasphaera_A;s__	98.42	0.54	3.75	57	67.9	0.446	0.436	0.507	1378316	2	47
TET2	GCA_020160795.1	d__Bacteria;p__Actinobacteriota;c__Actinobacteria;o__Actinomycetales;f__Dermatophilaceae;g__Tetrasphaera_A;s__Tetrasphaera_A	98.92	0.05	3.96	66	69.3	0.803	0.236	1.244	2538782	1	76
LEU1	GCA_020161315.1	d__Bacteria;p__Actinobacteriota;c__Actinobacteria;o__Actinomycetales;f__Microbacteriaceae;g__Leucobacter;s__	96.06	2.05	3.01	74	63.5	0.272	0.083	0.093	99061	3	47
LEU2	GCA_020161175.1	d__Bacteria;p__Actinobacteriota;c__Actinobacteria;o__Actinomycetales;f__Microbacteriaceae;g__Leucobacter;s__Leucobacter	83.22	1.48	2.31	140	64.8	0.065	0.101	0.092	22050	2	44
SAL1	GCA_020160915.1	d__Bacteria;p__Actinobacteriota;c__Actinobacteria;o__Actinomycetales;f__Microbacteriaceae;g__Salinibacterium;s__	97.81	0	2.93	8	67.2	0.335	0.142	0.559	178111	2	45
NANO1	GCA_020161245.1	d__Bacteria;p__Actinobacteriota;c__Actinobacteria;o__Nanopelagicales;f__;g__;s__	99.14	3.68	4.29	95	72.7	0.106	0.047	0.172	64510	1	52
PROP1	GCA_020161795.1	d__Bacteria;p__Actinobacteriota;c__Actinobacteria;o__Propionibacteriales;f__Propionibacteriaceae;g__;s__	91.04	0.91	3.47	67	69.3	0.063	0.108	0.206	100351	0	60
PROP2	GCA_020161755.1	d__Bacteria;p__Actinobacteriota;c__Actinobacteria;o__Propionibacteriales;f__Propionibacteriaceae;g__Propionicimonas;s__	93.63	3.02	4.08	61	70.7	0.094	0.046	0.413	130384	3	52
PROP3	GCA_020161015.1	d__Bacteria;p__Actinobacteriota;c__Actinobacteria;o__Propionibacteriales;f__Propionibacteriaceae;g__Propionicimonas;s__	94.14	3.15	3.67	65	71.6	0.074	0.176	0.249	96105	0	51
FIMBRI1	GCA_020161505.1	d__Bacteria;p__Armatimonadota;c__Fimbriimonadia;o__Fimbriimonadales;f__Fimbriimonadaceae;g__Uphvl-Ar1;s__	96.55	0	3.14	38	58.8	0.068	0.234	0.009	27830	3	48
BAC1	GCA_020161835.1	d__Bacteria;p__Bacteroidota;c__;o__;f__;g__;s__	94.52	0	4.40	36	41.6	0.345	0.024	0.003	32140	4	42
BAC2	GCA_020162035.1	d__Bacteria;p__Bacteroidota;c__Bacteroidia;o__AKYH767;f__b-17BO;g__;s__	99.05	0.48	3.17	31	29.6	0.757	0.010	0.015	46346	3	32
CHIT1	GCA_020161435.1	d__Bacteria;p__Bacteroidota;c__Bacteroidia;o__Chitinophagales;f__Chitinophagaceae;g__;s__	99.01	0	4.19	10	46.3	0.183	0.174	3.613	3141341	0	34
CHIT2	GCA_020161535.1	d__Bacteria;p__Bacteroidota;c__Bacteroidia;o__Chitinophagales;f__Chitinophagaceae;g__Flavihumibacter;s__	100	1.23	4.03	23	48.2	0.195	0.383	0.033	24003	3	40
SAP1	GCA_020160935.1	d__Bacteria;p__Bacteroidota;c__Bacteroidia;o__Chitinophagales;f__Saprospiraceae;g__;s__	96.53	0.99	5.84	51	50.3	0.226	0.007	0.128	702648	3	36
SAP2	GCA_020160855.1	d__Bacteria;p__Bacteroidota;c__Bacteroidia;o__Chitinophagales;f__Saprospiraceae;g__OLB8;s__	97.52	0.5	3.73	65	37.2	0.290	0.167	0.016	10636	3	34
LEAD1	GCA_020161355.1	d__Bacteria;p__Bacteroidota;c__Bacteroidia;o__Cytophagales;f__Spirosomaceae;g__Leadbetterella;s__	99.11	0.6	4.81	17	37.7	0.136	0.002	0.858	1017458	2	36
RUN1	GCA_020161055.1	d__Bacteria;p__Bacteroidota;c__Bacteroidia;o__Cytophagales;f__Spirosomaceae;g__Runella;s__Runella	100	0	7.44	60	44.4	0.124	1.088	1.749	10725342	2	40
FLAVO1	GCA_020161455.1	d__Bacteria;p__Bacteroidota;c__Bacteroidia;o__Flavobacteriales;f__Flavobacteriaceae;g__Flavobacterium;s__	99.29	0.35	3.08	18	32.5	0.030	0.002	0.742	3002991	3	36
CHRYS1	GCA_020161485.1	d__Bacteria;p__Bacteroidota;c__Bacteroidia;o__Flavobacteriales;f__Weeksellaceae;g__Chryseobacterium_A;s__Chryseobacterium_A	100	0.25	2.57	11	36.7	0.107	3.917	0.358	209940	2	35
BAC3	GCA_020162015.1	d__Bacteria;p__Bacteroidota;c__Bacteroidia;o__NS11–12g;f__UKL13-3;g__B1;s__	100	0	3.74	45	41.1	0.445	0.892	0.693	9991372	0	34
IGNAVI1	GCA_020161395.1	d__Bacteria;p__Bacteroidota;c__Ignavibacteria;o__Ignavibacteriales;f__Ignavibacteriaceae_A;g__UTCHB3;s__	97.27	0.55	4.07	21	42.2	0.163	0.635	0.025	58496	3	44
RTHERM1	GCA_020160835.1	d__Bacteria;p__Bacteroidota;c__Rhodothermia;o__Rhodothermales;f__;g__;s__	98.36	1.38	3.25	36	67	0.328	0.050	0.060	116472	3	52
ANAER1	GCA_020161935.1	d__Bacteria;p__Chloroflexota;c__Anaerolineae;o__SBR1031;f__A4b;g__;s__	98.17	0	7.64	32	54.2	0.375	0.190	0.153	910673	4	48
HERP1	GCA_020161265.1	d__Bacteria;p__Chloroflexota;c__Chloroflexia;o__Chloroflexales;f__Herpetosiphonaceae;g__Herpetosiphon;s__	99.09	0.91	6.04	13	50.2	0.774	0.025	0.008	7917	0	55
OBS1	GCA_020161235.1	d__Bacteria;p__Cyanobacteria;c__Vampirovibrionia;o__Obscuribacterales;f__Obscuribacteraceae;g__Obscuribacter;s__Obscuribacter	98.28	0.94	5.09	17	49.2	6.272	0.681	0.197	1713299	6	42
FUSI1	GCA_020161295.1	d__Bacteria;p__Firmicutes_A;c__Clostridia;o__Peptostreptococcales;f__Fusibacteraceae;g__UBA5201;s__	96.5	1.75	3.08	41	42.8	0.001	0.580	0.001	11649	3	57
GEMMA1	GCA_020161135.1	d__Bacteria;p__Gemmatimonadota;c__Gemmatimonadetes;o__Gemmatimonadales;f__Gemmatimonadaceae;g__;s__	98.35	3.3	4.55	8	70.1	0.004	0.031	0.494	2624259	3	55
SACCH1	GCA_020160975.1	d__Bacteria;p__Patescibacteria;c__Saccharimonadia;o__Saccharimonadales;f__Saccharimonadaceae;g__Saccharimonas;s__	84.48	0	0.97	1	49.6	0.637	1.437	0.035	65079	3	43
ALPHA1	GCA_020161965.1	d__Bacteria;p__Proteobacteria;c__Alphaproteobacteria;o__;f__;g__;s__	82.43	2.65	3.94	581	64.6	0.015	0.165	0.007	1283274	3	39
CAED1	GCA_020161545.1	d__Bacteria;p__Proteobacteria;c__Alphaproteobacteria;o__Caedimonadales;f__UBA1908;g__;s__	86.36	1.1	1.88	96	52.8	0.034	0.201	0.002	41264	3	35
BREV1	GCA_020161595.1	d__Bacteria;p__Proteobacteria;c__Alphaproteobacteria;o__Caulobacterales;f__Caulobacteraceae;g__Brevundimonas;s__Brevundimonas	97.51	3.41	3.07	155	67.2	0.011	0.254	0.004	27852	2	45
CAULO1	GCA_020161365.1	d__Bacteria;p__Proteobacteria;c__Alphaproteobacteria;o__Caulobacterales;f__Caulobacteraceae;g__Caulobacter;s__	100	0	4.43	25	66.9	0.048	0.093	0.589	4627825	3	55
HYPHO1	GCA_020161405.1	d__Bacteria;p__Proteobacteria;c__Alphaproteobacteria;o__Caulobacterales;f__Hyphomonadaceae;g__UBA1942;s__	98.43	0.32	2.98	6	39.4	0.844	0.006	2.208	4138107	0	33
REYR1	GCA_020160955.1	d__Bacteria;p__Proteobacteria;c__Alphaproteobacteria;o__Reyranellales;f__Reyranellaceae;g__Reyranella;s__	89.96	7.34	5.08	210	70	0.057	0.090	0.238	224063	3	53
REYR2	GCA_020160995.1	d__Bacteria;p__Proteobacteria;c__Alphaproteobacteria;o__Reyranellales;f__Reyranellaceae;g__Reyranella;s__Reyranella	91.04	6.01	5.71	258	65.3	0.074	0.102	0.134	62018	1	53
ANDERS1	GCA_020161855.1	d__Bacteria;p__Proteobacteria;c__Alphaproteobacteria;o__Rhizobiales;f__Anderseniellaceae;g__PALSA-927;s__	97.64	0.4	3.36	19	61.6	0.187	0.175	0.029	25238	2	46
BEIJ1	GCA_020161915.1	d__Bacteria;p__Proteobacteria;c__Alphaproteobacteria;o__Rhizobiales;f__Beijerinckiaceae;g__Bosea;s__	81.6	8.48	4.44	777	66.3	0.156	0.319	0.423	338238	0	43
BEIJ2	GCA_020161975.1	d__Bacteria;p__Proteobacteria;c__Alphaproteobacteria;o__Rhizobiales;f__Beijerinkiaceae_A;g__;s__	81.18	5.25	3.99	465	62.5	0.042	0.157	0.018	28432	0	42
BEIJ3	GCA_020161475.1	d__Bacteria;p__Proteobacteria;c__Alphaproteobacteria;o__Rhizobiales;f__Beijerinkiaceae_A;g__PAR1;s__	76.21	1.72	3.08	320	63.3	0.017	1.744	0.099	77102	0	41
BEIJ4	GCA_020161575.1	d__Bacteria;p__Proteobacteria;c__Alphaproteobacteria;o__Rhizobiales;f__Beijerinkiaceae_A;g__PAR1;s__	97.89	0	3.19	17	63.2	0.176	0.538	0.014	26820	0	47
PHREA1	GCA_020161695.1	d__Bacteria;p__Proteobacteria;c__Alphaproteobacteria;o__Rhizobiales;f__Phreatobacteraceae;g__Phreatobacter;s__	98.35	3.96	4.69	38	67.7	0.022	0.273	0.103	133243	1	50
RHIZO1	GCA_020161035.1	d__Bacteria;p__Proteobacteria;c__Alphaproteobacteria;o__Rhizobiales;f__Rhizobiaceae;g__Aminobacter;s__Aminobacter	94.26	5.5	5.50	80	63.8	0.136	0.095	0.095	219213	3	48
RHIZO2	GCA_020161665.1	d__Bacteria;p__Proteobacteria;c__Alphaproteobacteria;o__Rhizobiales;f__Rhizobiaceae;g__QFOR01;s__	88.41	2.12	3.39	43	60.6	0.035	0.335	0.003	24536	0	47
RHIZO3	GCA_020161625.1	d__Bacteria;p__Proteobacteria;c__Alphaproteobacteria;o__Rhizobiales;f__Rhizobiaceae;g__Shinella;s__Shinella	78.53	6.03	6.98	935	63.6	0.010	0.169	0.037	149921	0	48
RHODO1	GCA_020161655.1	d__Bacteria;p__Proteobacteria;c__Alphaproteobacteria;o__Rhodobacterales;f__Rhodobacteraceae;g__Defluviimonas;s__	100	0.35	4.08	24	65.5	0.321	0.141	0.848	3645270	0	44
RHODO2	GCA_020161615.1	d__Bacteria;p__Proteobacteria;c__Alphaproteobacteria;o__Rhodobacterales;f__Rhodobacteraceae;g__Pararhodobacter;s__	99.09	1.19	4.87	26	67.9	0.084	0.534	0.009	25807	1	49
RHODO3	GCA_020160875.1	d__Bacteria;p__Proteobacteria;c__Alphaproteobacteria;o__Rhodospirillales_C;f__Rhodospirillaceae_A;g__;s__	91.2	2.27	3.76	236	62.2	0.153	0.046	0.185	153017	1	39
RICK1	GCA_020160775.1	d__Bacteria;p__Proteobacteria;c__Alphaproteobacteria;o__Rickettsiales;f__Rickettsiaceae;g__GCA-2402195;s__	75.59	1.58	1.18	82	34.5	0.085	0.075	0.052	17671	2	26
SPHING1	GCA_020160755.1	d__Bacteria;p__Proteobacteria;c__Alphaproteobacteria;o__Sphingomonadales;f__Sphingomonadaceae;g__Sphingopyxis;s__	99.98	1.56	4.31	20	65.1	0.026	0.014	0.607	600695	3	47
ALIC1	GCA_020161945.1	d__Bacteria;p__Proteobacteria;c__Gammaproteobacteria;o__Burkholderiales;f__Burkholderiaceae;g__Alicycliphilus;s__	99.64	1.04	3.83	33	66.3	0.166	2.959	0.738	770970	1	48
OTTO1	GCA_020161215.1	d__Bacteria;p__Proteobacteria;c__Gammaproteobacteria;o__Burkholderiales;f__Burkholderiaceae;g__Ottowia;s__	93.66	5.56	4.52	250	67.1	0.011	0.276	0.004	26717	1	46
OTTO2	GCA_020161715.1	d__Bacteria;p__Proteobacteria;c__Gammaproteobacteria;o__Burkholderiales;f__Burkholderiaceae;g__Ottowia;s__Ottowia	99.26	0.62	3.40	35	69.1	0.372	4.140	0.424	121379	1	50
RAM1	GCA_020161775.1	d__Bacteria;p__Proteobacteria;c__Gammaproteobacteria;o__Burkholderiales;f__Burkholderiaceae;g__Ramlibacter;s__	99.84	0.06	4.36	32	66.1	0.778	0.536	1.814	1832037	1	45
RUBRI1	GCA_020161065.1	d__Bacteria;p__Proteobacteria;c__Gammaproteobacteria;o__Burkholderiales;f__Burkholderiaceae;g__Rubrivivax;s__	99.52	0.05	6.29	41	71.2	0.236	0.347	0.306	1259737	1	73
VITREO1	GCA_020161145.1	d__Bacteria;p__Proteobacteria;c__Gammaproteobacteria;o__Burkholderiales;f__Burkholderiaceae;g__Vitreoscilla_A;s__	100	0.7	3.51	13	68.9	0.397	4.498	0.530	382529	1	46
CAPIA	NA	d__Bacteria;p__Proteobacteria;c__Gammaproteobacteria;o__Burkholderiales;f__Rhodocyclaceae;g__Accumulibacter;s__Accumulibacter	100	0.03	4.59	61	63.8	18.797	10.533	0.106	2411395	0	46
CAPIIA	NA	d__Bacteria;p__Proteobacteria;c__Gammaproteobacteria;o__Burkholderiales;f__Rhodocyclaceae;g__Accumulibacter;s__Accumulibacter	99.84	0.24	4.64	81	64.3	33.479	26.824	49.334	102762132	0	44
ZOO1	GCA_020161115.1	d__Bacteria;p__Proteobacteria;c__Gammaproteobacteria;o__Burkholderiales;f__Rhodocyclaceae;g__Zoogloea;s__	91.62	3.51	4.99	501	65.7	0.090	0.026	0.106	913411	4	59
LEG1	GCA_020161725.1	d__Bacteria;p__Proteobacteria;c__Gammaproteobacteria;o__Legionellales;f__Legionellaceae;g__;s__	92.74	1.07	2.58	182	36.1	0.094	0.126	0.006	19591	1	27
LUTEI1	GCA_020161335.1	d__Bacteria;p__Proteobacteria;c__Gammaproteobacteria;o__Xanthomonadales;f__Xanthomonadaceae;g__Luteimonas;s__	96.89	0.71	3.56	252	69.9	0.002	0.309	0.011	49418	1	39
PSEUDO1	GCA_020160895.1	d__Bacteria;p__Proteobacteria;c__Gammaproteobacteria;o__Xanthomonadales;f__Xanthomonadaceae;g__Pseudoxanthomonas_A;s__	99.95	0.89	3.67	28	67.8	0.416	0.730	3.125	3964795	2	50
PSEUDO2	GCA_020161075.1	d__Bacteria;p__Proteobacteria;c__Gammaproteobacteria;o__Xanthomonadales;f__Xanthomonadaceae;g__Pseudoxanthomonas;s__	99.02	0	2.99	6	69.6	1.750	6.111	1.228	515369	3	52

Genome code names match names used in all figures and within the text. Classifications were assigned using the GTDB-tk [87] and confirmed by comparing against select publicly available references and a subset of HQ MAGs from Singleton et al. 2021 [40]. Completeness and redundancy estimates and GC content were calculated by CheckM [43]. tRNA and rRNA predictions were performed with Barrnap as part of the Prokka software [88]. Relative abundance estimates reflect the proportion of reads mapped to the genome in that sample divided by the total number of reads mapped to all genomes as performed with SingleM. Table available at https://figshare.com/articles/dataset/EBPR_SBR_MAGs_Metadata/13063874.

Each bin was functionally annotated using the KEGG database through an HMM-based approach under KEGG release 93.0 using the command-line KofamKOALA pipeline [44, 45], selecting annotations that were significant hits above the specific HMM threshold. This resulted in 117,657 total annotations with 5,228 unique annotations. We used a metatranscriptomic dataset of six timepoints collected over a single EBPR cycle from Oyserman et al. 2016 [42], with three timepoints from the anaerobic phase and three from the aerobic phase. Raw reads were quality filtered using BBtools suite v38.07 [46] and ribosomal rRNA was removed from each sample using SortMeRNA [47]. Reads from each sample were mapped against the concatenated set of open reading frames from all 66 bins using kallisto v0.44.0 and parsed using the R package tximport [48, 49].

### TbasCO method implementation

The TbasCO package identifies expression-based attributes of predefined traits using time-series (meta)transcriptomics data (Fig. 1). As expression patterns are determined by the time-points assessed in an experiment, it is important to design the sampling regime to capture relevant ecophysiological changes within the ecosystem. In general, traits are defined by the presence of a pathway or other collection of genes from an externally provided database. A weighted distance metric between expression patterns for all genes that define a trait is calculated, and statistically significant similarity is determined based on the background distribution of a trait of equal size. Thereby, two or more organisms with a statistically similar expression pattern for a trait share an *attribute*. As the expression profiles of genes within a trait are compared across genomes independently, co-expression of genes within a genome is not a pre-requisite for identifying an attribute.

#### Input and preprocessing

The input that is accepted by TbasCO is a table of RNAseq counts in csv format. Each row is treated as gene that has columns for the gene/locus name, counts per sample, the genome the gene belongs to, and the KEGG Orthology (KO) identifier. The RNAseq counts table may be provided pre-normalized or can be normalized by the program. The default normalization method is designed to minimize compositional bias in the differential abundance and activity of constituent populations in metatranscriptomics studies. RNA expression counts are therefore normalized relative to each genomic bin separately for each sample [42]. After normalization, a pruning step is introduced to filter genes that have zero counts or a mean absolute deviation of less than one across all time points. To make inter-organismal comparisons of the relative contribution of a gene to total measured organismal RNA, an additional statistic is calculated ranking the expression counts from each sample from highest to lowest. The ranks for each sample are then normalized by dividing them by the maximum rank value in that sample. This normalization is applied to make ranks comparable between organisms with different genome sizes.

To assess the statistical significance of the calculated distances between the expression patterns of all genes within a trait, random background distributions are created for (1) individual genes and (2) traits of N genes. For individual genes, three different distributions were calculated, based on the distances between randomly sampled open reading frames, randomly sampled genes with an annotation (but not necessarily the same annotation), and randomly sampled genes with the same annotation. The background distribution for a trait of N genes is based on the distances between randomly composed sets of genes. For each gene pair, two distances metrics are calculated, the Pearson Correlation (PC) and the Normalized Rank Euclidean Distance (NRED). In practice, it is often found that a certain annotation is assigned to multiple genes in the same genome. If this occurs, there is an option to use either a random selection, or the highest scoring pair. In the latter case, a correction for multiple testing is implemented. This process is repeated N-times, where N equals the number of genes in any given trait. The background distribution for traits is determined by first randomly sampling two genomes, identifying the overlap in annotations, and finally artificially defining a trait containing N annotations. For each annotation in the trait, the distances are calculated between genome A and genome B, as described in the previous section. As modules vary in size, this process is repeated for traits of different sizes.

#### Identifying attributes

TbasCO provides both a cluster-based and pair-wise approach to identify attributes. In both methods, the distance between expression patterns of a trait between two genomes is first calculated based on a composite Z score of the PC and NRED for each gene composing the trait. In the cluster-based analysis, the distances are subsequently clustered using the Louvain clustering algorithm to identify trait attributes. To determine if an attribute is significantly similar or not, a one-sided T-test between the attribute and the random background distribution of traits is conducted. This is done for both cluster-based and model-based comparisons. Many traits are complex and represented in databases such as KEGG by numerous alternative routes. To deal with this complexity, each pathway is expanded into all possible alternative routes. Due to the extremely high number of alternative routes for some traits, attributes are pruned based on a strict requirement of 100% completion.

#### Distance calculations

To determine the similarity in expression patterns between genes, two dissimilarity metrics are calculated: the PC between RNAseq counts across samples, and the NRED, where ranks are a measure of relative abundance of RNA in each sample, normalized the abundance of RNA in the corresponding genome. These distance scores are converted to Z scores using a background distribution of distances between randomly sampled genes as previously described. To determine statistically significant similarities in expression patterns of a trait, a composite score is calculated. For each of these genes the PC and NRED are calculated and transformed to Z scores and combined as (−1*PC + NRED). The distance of the trait between two genomes is defined as the average of these composite distance scores. If traits being compared do not have 100% overlap in gene content, then the dissimilarity score is normalized by the Jaccard distance between gene content of the trait.

$$\left( { - PC + NRED} \right) \ast \left( {1 - dJ} \right)$$

#### Statistical assessment of trait attributes

In both model-based and pair-wise approaches, the distance is first calculated between expression patterns of a trait between two genomes based on the composite Z score of the PC and NRED for each gene composing the trait. In the clustering-based analysis, the distances are subsequently clustered using the Louvain clustering algorithm to identify trait-attributes. To determine if attributes are significantly similar, a one-sided T-test is conducted between the attribute and a background distribution of randomly sampled traits with the same number of genes. To derive the random background distributions, multiple distributions are calculated ranging in gene numbers from the smallest trait to the largest trait in the dataset as described previously. For each background distribution, N (default: 10,000) traits are randomly composed. The distances between these artificial traits are calculated in the same way as for the actual traits. In addition to a statistical pruning step, the attributes are pruned based on a strict requirement of 100% completion of each module. A benchmarking analysis to examine the effects of different parameters, including the presence of zero counts, was conducted to determine their influence on the number of attributes identified and may be found in the supplementary materials (Supplementary Table 1, Supplementary Figs. 2–4).

## Results and discussion

### Reconstructing a diverse EBPR SBR community

**Fig. 2:**
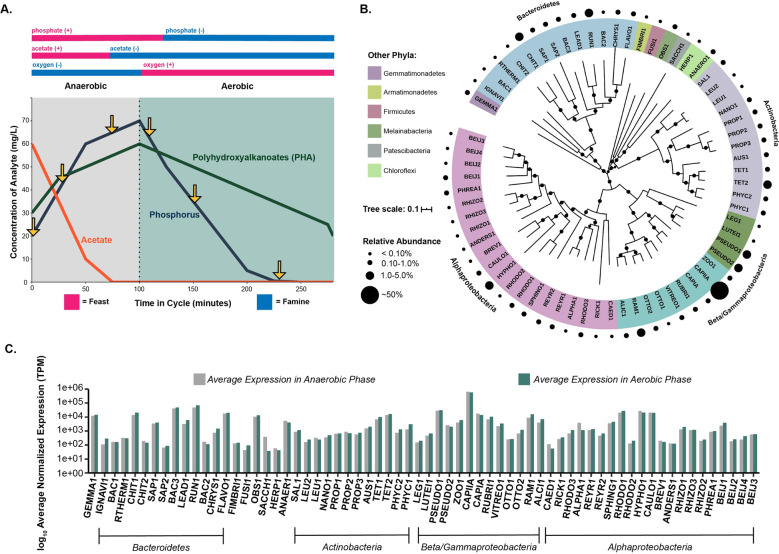
Genome-resolved metatranscriptomics approach of an EBPR system. Application of a genome-resolved metatranscriptomics approach to a lab-scale sequencing batch reactor (SBR) designed to enrich for Accumulibacter. **A** Schematic of the main cycle parameters and analyte dynamics of an SBR simulating EBPR. Six samples were taken for RNA sequencing within the cycle at time-points denoted by arrows. **B** Phylogenetic identity and abundance patterns of 66 assembled MAGs from the EBPR system. The phylogenetic tree was constructed from concatenated markers contained in the GTDB-tk with muscle, calculated with RAxML, and visualized in iTOL. A phylogenetic tree of all 66 MAGs with reference genomes and high-quality genomes from Singleton et al. constructed with concatenated markers from GTDB-tk are provided in Supplementary Fig. 1. Sizes of circles represent relative abundance patterns calculated from metagenomic reads obtained from a sample collected the same day as the metatranscriptomic experiment was performed, and are not to scale. **C** Transcriptional patterns of each MAG in the anaerobic and aerobic phases of the EBPR cycle. RNA-seq reads from each time-point were competitively mapped to all 66 assembled MAGs and counts normalized by transcripts per million (TPM). Total counts in the anaerobic and aerobic phases for each genome were averaged separately and plotted on a log scale. Order of MAGs from left to right mirrors the order of MAGs in the phylogenetic tree in **B** from the top of the circle going clockwise.

To explore trait-based transcriptional dynamics of a semi-complex microbial community, we applied genome-resolved metagenomics and metatranscriptomics to an EBPR sequencing-batch reactor (SBR) ecosystem (Fig. 2). We previously performed a metatranscriptomics time-series experiment over the course of a normally operating EBPR cycle to investigate the regulatory controls of Accumulibacter gene expression [42]. In this experiment, six samples were collected for RNA sequencing: three from the anaerobic phase and three from the aerobic phase (Fig. 2A). Additionally, three metagenomes were collected from the same month of the metatranscriptomic experiment, including a sample from the same date of the experiment. We reassembled contemporary Accumulibacter clade IIA and IA genomes that were previously assembled from the same bioreactor system [27, 28]. The genomes of Accumulibacter clades IA and IIA are similar by approximately 85% average-nucleotide identity [28, 31], which is well below the common species-resolved cutoff of 95%, and these groups have recently been designated as separate species (*Candidatus* Accumulibacter regalis and *Candidatus* Accumulibacter phosphatis, respectively) [35]. However, we maintain references to the Accumulibacter clade nomenclature based on polyphosphate kinase (*ppk1*) sequence identity throughout the manuscript (CAPIA and CAPIIA) [31, 50, 51]. During the experiment, the bioreactor was highly enriched in Accumulibacter clade IIA, accounting for approximately 50% of the mapped metagenomic reads and the highest transcriptional counts (Fig. 2B, C) [42]. Whereas Accumulibacter clade IA exhibited low abundance patterns but was within the top 10 genomes with the highest total transcriptional counts (Fig. 2C).

Although this bioreactor system was highly enriched in Accumulibacter, a diverse bacterial community persisted and was active in this ecosystem (Fig. 2B, C). We reconstructed representative population genomes of the microbial community of the SBR system, resulting in 64 metagenome-assembled genomes (MAGs) of the (non-Accumulibacter) bacterial community. Interestingly, we recovered genomes of experimentally verified and putative PAOs previously not detected in these bioreactors, including two *Tetrasphaera spp*. (TET1 and TET2) ‘*Candidatus Obscuribacter phosphatis’* (OBS1), and *Gemmatimonadetes* (GEMMA1). Pure cultures of *Tetrasphaera* have been experimentally shown to cycle polyphosphate without incorporating PHA [37], deviating from the hallmark Accumulibacter PAO model. The first cultured representative of the *Gemmatimonadetes* phylum *Gemmatimonas aurantiaca* was isolated from an SBR simulating EBPR and was shown to accumulate polyphosphate through Neisser and DAPI staining [52]. Additionally, *Ca. Obscuribacter phosphatis* has been hypothesized to cycle phosphorus based on the presence of genes for phosphorus transport, polyphosphate incorporation, and potential for both anaerobic and aerobic respiration [38], and was enriched in a photobioreactor EBPR system [53]. Both *Tetrasphaera spp*. TET1 and TET2, OBS1, and GEMMA1 groups exhibit higher relative abundance patterns than CAPIA but have similar relative transcriptional levels (Fig. 2B, C, Table 1).

Numerous SBR MAGs among the *Actinobacteria* and *Proteobacteria* contain the high-affinity phosphorus transporter *pstABCS* system, polyphosphate kinase *ppk1*, and the low-affinity *pit* phosphorus transporter (Supplementary Fig. 5). Additionally, select MAGs within the *Alphaproteobacteria*, *Betaproteobacteria*, and *Gammaproteobacteria* contain all required subunits for polyhydroxyalkanoate synthesis (Supplementary Fig. 5). Other abundant and transcriptionally active groups in the SBR ecosystem that are not predicted to be PAOs are members of the *Bacteroidetes* such as CHIT1 within the *Chitinophagaceae*, and *Cytophagales* members *Runella* sp. RUN1 and *Leadbetterella* sp. LEAD1 (Fig. 2B, C, Table 1). Interestingly, an uncharacterized group within the *Bacteroidetes*, represented by BAC1, contributed the third most to the pool of transcripts (Fig. 2C), and did not show phylogenetic similarity to MAGs assembled from Danish full-scale wastewater treatment systems [40] (Supplementary Fig. 1). Other groups from which we assembled MAGs for that do not exhibit clear roles in EBPR systems were *Chloroflexi* ANAER1 and HERP1 MAGs, *Armatimonadetes* FIMBRI1, *Firmicutes* FUSI1, and *Patescibacteria* SACCH1. Members of the *Chloroflexi* are filamentous bacteria that have been associated with bulking and foaming events in full-scale WWTPs [54–56], but also aid in forming the scaffolding around floc aggregates and degrade complex polymers [56–58]. The *Patescibacteria* (formerly TM7) are widespread but low abundant members of natural and engineered ecosystems, have reduced genome sizes, and may contribute to filamentous bulking in activated sludge [22, 59]. To summarize, lab-scale SBRs designed to enrich for Accumulibacter contain diverse bacterial microorganisms [27, 32], but their ecological functions and putative interactions remain to be fully understood in the context of the EBPR ecosystem.

### Identifying expression-based trait attributes among the EBPR SBR community with TbasCO

**Fig. 3:**
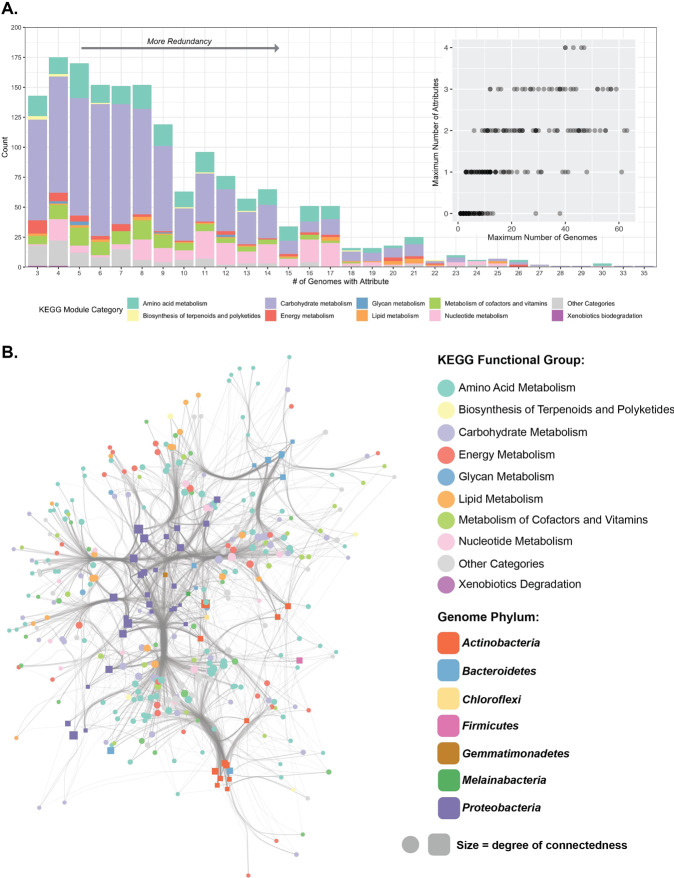
Clustering and distribution of trait attributes across EBPR SBR community members. Using the TbasCO method, we identified expression-based trait attributes from predefined trait modules in the KEGG library and explored the distribution of these trait attributes across community members. **A** Distribution of trait-attributes among sets of genomes. Bars represent the number of trait-attributes present in a set number of genomes and colored by KEGG module category. Among a total of 35 genomes, trait attributes present between 3 and 18 genomes are designated as niche differentiating, whereas trait attributes present in 19 or greater genomes are designated as core trait attributes. Inset figure demonstrates the maximum number of attributes for the maximum number of genomes. **B** Cytoscape network showing the connectedness of genomes to trait attributes. The network was filtered to only include nodes with more than 5 connections, therefore filtering out both genomes with few trait attributes and trait attributes connected to less than 5 genomes. Genomes are represented as squares colored by phylum, and trait attributes are represented as circles colored by KEGG category. The size of both the squares and circles represents the number of connections to that genome or trait attribute, respectively.

Current metatranscriptomics analyses often employ either a gene-centric [31, 60–62] or genome-centric approach [42, 63–65]. In both approaches, highly, differentially, or co-expressed genes are identified and tested for enrichment of specific functions. Enrichment- or annotation-based approaches are employed in numerous metatranscriptomics tools such as MG-RAST, MetaTrans, SAMSA2, COMAN, IMP, and Anvi’o [66–71]. Here, we expand on the use of molecular markers as traits by defining expression attributes by leveraging *a priori* knowledge from predefined trait libraries, such as the KEGG database [72], to statistically assess inter-species expression patterns of genes that together form a trait (Fig. 1). First, our results showed that there is statistically significant transcriptional conservation of genes at the community level; genes that share an annotation were significantly more similar than expected using two different distance metrics (NRED: *p* value <2.2e–16, PC: *p* value <2.2e–16). Extending this statistical analysis to the trait level, we identified 1674 attributes distributed across the 66 genomes. On average, we identified 9.12 genomes per attribute (SD -5.22), with a minimum of 3 genomes and a maximum of 35 (Fig. 3B). Based on these statistics, we defined redundant attributes as those two standard deviations above the mean (19 genomes). With this cutoff applied, we identified 79 redundant trait attributes mostly belonging to pathways among carbohydrate metabolism, purine metabolism, and fatty acid metabolism categories (Table 2). Of 290 traits, we identified 97 traits with two or more attributes identified (33%). Of these, traits in 10 or more genomes were twice as likely to have two or more attributes (65%), suggesting that divergent expression patterns for a trait are common, and may represent a niche-differentiating feature (Fig. 3A). Henceforth, when multiple attributes are identified for a trait, we refer to these as niche-differentiating attributes.

**Table 2:** KEGG pathways for core trait-attributes present in greater than 19 genomes.

Module description	Number of attributes
Citrate cycle, second carbon oxidation, 2-oxoglutarate => oxaloacetate [PATH:map00020 map01200 map01100]	13
Citrate cycle (TCA cycle, Krebs cycle) [PATH:map00020 map01200 map01100]	10
Shikimate pathway, phosphoenolpyruvate + erythrose-4P = > chorismate [PATH:map00400 map01230 map01100 map01110]	8
Fatty acid biosynthesis, initiation [PATH:map00061 map01212 map01100]	7
Glycolysis, core module involving three-carbon compounds [PATH:map00010 map01200 map01230 map01100]	7
Adenine ribonucleotide biosynthesis, IMP = > ADP,ATP [PATH:map00230 map01100]	4
Guanine ribonucleotide biosynthesis IMP = > GDP,GTP [PATH:map00230 map01100]	4
Inosine monophosphate biosynthesis, PRPP + glutamine => IMP [PATH:map00230 map01100]	4
Isoleucine biosynthesis, threonine => 2-oxobutanoate => isoleucine [PATH:map00290 map01230 map01100]	3
NADH:quinone oxidoreductase, prokaryotes [PATH:map00190]	3
beta-Oxidation, acyl-CoA synthesis [PATH:map00061 map00071 map01212 map01100]	2
F-type ATPase, prokaryotes and chloroplasts [PATH:map00190 map00195]	2
Valine/isoleucine biosynthesis, pyruvate => valine / 2-oxobutanoate => isoleucine [PATH:map00290 map00770 map01210 map01230 map01100 map01110]	2
CAM (Crassulacean acid metabolism), dark [PATH:map00620 map00710 map01200 map01100 map01120]	1
Cytochrome c oxidase, cbb3-type [PATH:map00190]	1
Cytochrome c oxidase, prokaryotes [PATH:map00190]	1
dTDP-L-rhamnose biosynthesis [PATH:map00521 map00523 map01100 map01130]	1
Leucine biosynthesis, 2-oxoisovalerate => 2-oxoisocaproate [PATH:map00290 map01210 map01230 map01100 map01110]	1
Phosphatidylethanolamine (PE) biosynthesis, PA = > PS = > PE [PATH:map00564 map01100]	1
PRPP biosynthesis, ribose 5 P = > PRPP [PATH:map00030 map00230 map01200 map01230 map01100]	1
Pyruvate oxidation, pyruvate => acetyl-CoA [PATH:map00010 map00020 map00620 map01200 map01100]	1
Semi-phosphorylative Entner-Doudoroff pathway, gluconate => glycerate-3P [PATH:map00030 map01200 map01100 map01120]	1
Threonine biosynthesis, aspartate => homoserine => threonine [PATH:map00260 map01230 map01100 map01110]	1

From the ecosystem perspective, a clear phylogenetic signal is observed in the distribution of attributes, as genomes cluster together by shared trait attributes by phylum with some exceptions, such as genomes belonging to the *Bacteroidetes, Actinobacteria*, and *Proteobacteria* clustering together, respectively (Fig. 3C). For simplicity, we filtered the network to only include nodes with more than 5 connections. Highly redundant trait attributes belonged to modules in the lipid metabolism, energy metabolism, and nucleotide metabolism KEGG functional categories. In contrast, more specialized trait attributes on the periphery of the network or amongst group-specific clusters such as within the *Actinobacteria* or subsets of the *Proteobacteria* belonged to amino acid metabolism, biosynthesis of terpenoids and polyketides, metabolism of cofactors and vitamins, and carbohydrate metabolism KEGG modules. Pathways of note that showed a high level of redundancy include the TCA cycle, isoleucine biosynthesis, acyl-CoA synthesis, threonine biosynthesis, and cytochrome c oxidase activity (Table 2). Large pathways with hundreds of possible routes such as glycolysis, the TCA cycle, gluconeogenesis, and the pentose phosphate pathway are not included in the main network and are displayed as individual networks (Supplementary Fig. 6).

We next explored the distribution of non-redundant attributes (e.g. 3–18 genomes) (Fig. 3B). A total of 796 trait attributes with low redundancy were identified belonging to pathways involved in carbohydrate cofactor and vitamin metabolism including glycolysis, gluconeogenesis, parts of the TCA cycle, tetrahydrofolate biosynthesis, tryptophan biosynthesis, and the pentose phosphate pathway (Table 3). Different sets of low redundancy trait attributes were identified within respective phyla (Supplementary Fig. 7). Between genomes belonging to the *Actinobacteria*, *Alphaproteobacteria, Bacteroidetes, Betaproteobacteria*, and *Gammaproteobacteria*, low redundancy attributes (belonging to less than half of the total genomes within the phylum) include carbohydrate metabolism, amino acid metabolism and metabolism of cofactors and vitamins (Supplementary Fig. 7). Redundant trait attributes within individual phyla belong to core energy metabolism pathways, fatty acid biosynthesis, and carbohydrate metabolism. However, even within individual phyla, non-redundant attributes include different amino acids and cofactors (Extended Table 1 - available on Figshare https://figshare.com/articles/dataset/Lineage-Specific_Core_and_Niche_Differentiating_Traits/15001200).

**Table 3:** KEGG Pathways for differentiating trait-attributes present between 3 and 18 genomes.

Module_description	Number of attributes
Glycolysis (Embden-Meyerhof pathway), glucose => pyruvate [PATH:map00010 map01200 map01100]	279
Citrate cycle (TCA cycle, Krebs cycle) [PATH:map00020 map01200 map01100]	208
Gluconeogenesis, oxaloacetate => fructose-6P [PATH:map00010 map00020 map01100]	76
Inosine monophosphate biosynthesis, PRPP + glutamine => IMP [PATH:map00230 map01100]	45
Citrate cycle, second carbon oxidation, 2-oxoglutarate => oxaloacetate [PATH:map00020 map01200 map01100]	31
Heme biosynthesis, plants and bacteria, glutamate => heme [PATH:map00860 map01100 map01110]	27
Tetrahydrofolate biosynthesis, GTP = > THF [PATH:map00790 map00670 map01100]	25
Tryptophan biosynthesis, chorismate => tryptophan [PATH:map00400 map01230 map01100 map01110]	25
Ornithine biosynthesis, glutamate => ornithine [PATH:map00220 map01210 map01230 map01100]	24
Histidine biosynthesis, PRPP = > histidine [PATH:map00340 map01230 map01100 map01110]	17
Pentose phosphate pathway (Pentose phosphate cycle) [PATH:map00030 map01200 map01100 map01120]	16
Lysine biosynthesis, succinyl-DAP pathway, aspartate => lysine [PATH:map00300 map01230 map01100]	12
Uridine monophosphate biosynthesis, glutamine (+ PRPP) = > UMP [PATH:map00240 map01100]	11

As noted previously, one of the most striking findings is that a majority, 65% of traits present in 10 or more genomes have multiple expression attributes. Thus, it seems that while the presence of marker genes suggests many organisms share a particular trait, the presence of niche-differentiating expression profiles suggest an alternative story, that there is a level of hidden metabolic diversity. For example, central carbon metabolism and energy pathways such as the TCA cycle, glycolysis, gluconeogenesis, and the pentose phosphate pathway are oftentimes considered core traits when only analyzing the presence and/or absence of individual markers belonging to these pathways. Among over 1000 high-quality MAGs assembled from full-scale Danish WWTPs, the TCA cycle and pentose phosphate pathway are highly represented among the abundant microorganisms, with glycolysis less so [40]. Whereas the TCA cycle and pentose phosphate pathway are present among a high number of genomes in the EBPR SBR community, different routes or parts of these pathways have niche-differentiating distributions (Supplementary Fig. 6, Tables 2 and 3). These finer-scale differences in expression of “core” traits may explain the persistence of a diverse community when solely fed acetate, as different lineages could employ similar carbon utilization pathways differently or in more versatile ways. Another salient aspect of this analysis is the astonishingly high number of possible routes within individual pathways here represented by their Disjunctive Normal Forms. For example, accounting for all alternative routes and enzymes, the glycolysis pathway has 100 s of possible routes. Layering upon this many expression attributes reveals a large hidden metabolic versatility.

### Dimensionality of the high-affinity phosphorus transporter system *PstABCS*

**Fig. 4:**
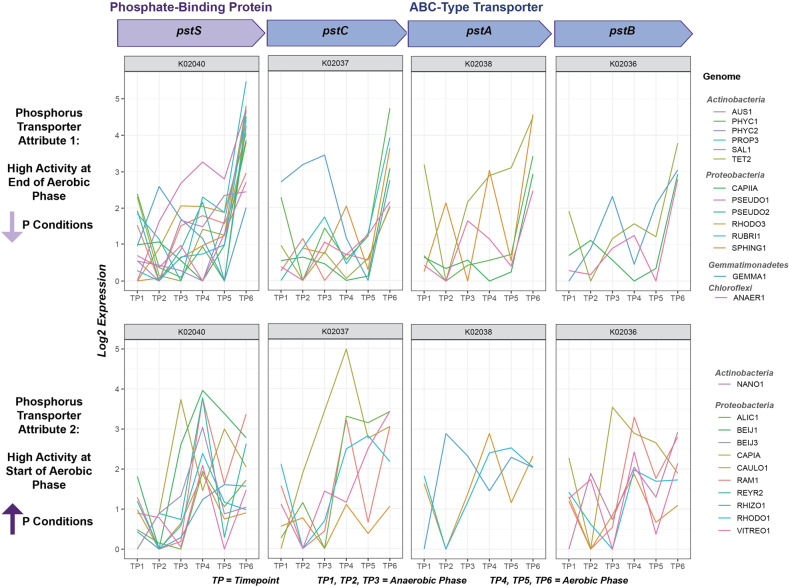
Trait attributes of the high-affinity phosphorus transporter system *pstABCS*. Using the TbasCO method, two trait attributes of the high-affinity phosphorus transporter system *pstABCS* were identified. The *pstABCS* system consists of a phosphate-binding protein and ABC-type transporter, and the corresponding KEGG orthologs for each subunit are shown. Timepoints 1–3 refer to the three anaerobic phase timepoints, and timepoints 4–6 refer to the three anaerobic phase timepoints (Fig. 1). Expression values are log-transformed based on setting the lowest expression value within each genome across the time-series to 0 for each subunit. Specific subunits for some genomes in both attributes are missing to the high cutoff thresholds for annotations. However we kept genomes with 2/4 subunits to show similarities in expression profiles. The first *pstABCS* trait-attribute includes microbial lineages that exhibited the highest expression of all subunits towards the end of the aerobic cycle, when phosphate concentrations are expected to be lowest. This includes microbial lineages within the *Actinobacteria, Proteobacteria, Gemmatimonadetes*, and *Chloroflexi*. The second *pstABCS* trait-attribute includes lineages that exhibited highest expression of all subunits upon the switch from anaerobic to aerobic phases, or when phosphate concentrations are expected to be the highest. This includes lineages within the *Actinobacteria* and *Proteobacteria*.

The EBPR ecosystem is characterized by its highly dynamic phosphorus cycles. To explore how different lineages respond to fluctuating phosphorus concentrations, we examined the expression-based attributes for the KEGG module of the high-affinity phosphorus transporter *pstABCS* (Fig. 4). The *pstABCS* system is an ABC-type transporter that strongly binds phosphate with high affinity under phosphorus-limiting conditions, and therefore we expected that the highest expression levels would be at the end of the aerobic cycle [73]. In contrast, we found that *pstABCS* expression was characterized by two different trait attributes. In the first attribute shared by 14 community members, all *pstABCS* components displayed the highest activity towards the end of the aerobic cycle, when phosphorus concentrations were depleted (Fig. 4, Attribute 1). Conversely, 11 community members displayed an alternate attribute where the highest activity of *pstABCS* was at the transition from anaerobic to aerobic phases when phosphorus concentrations are highest (Fig. 4, Attribute 2).

Interestingly, the two Accumulibacter clades IA and IIA are split amongst these separate *pstABCS* attributes. These results are in agreement with previous results showing that Accumulibacter clade IIC has a canonical *pstABCS* expression pattern (as in Fig. 4, Attribute 1), whereas the Accumulibacter clade IA has a non-canonical expression (as in Fig. 4, Attribute 2) [31]. By assigning trait attributes, we can extend these findings beyond Accumulibacter to other community members in the SBR ecosystem suggesting that there are conserved ecological pressures driving niche differentiating expression patterns in *pstABCS* within the EBPR community.

### Distribution and expression of truncated denitrification steps among EPBR community members

**Fig. 5:**
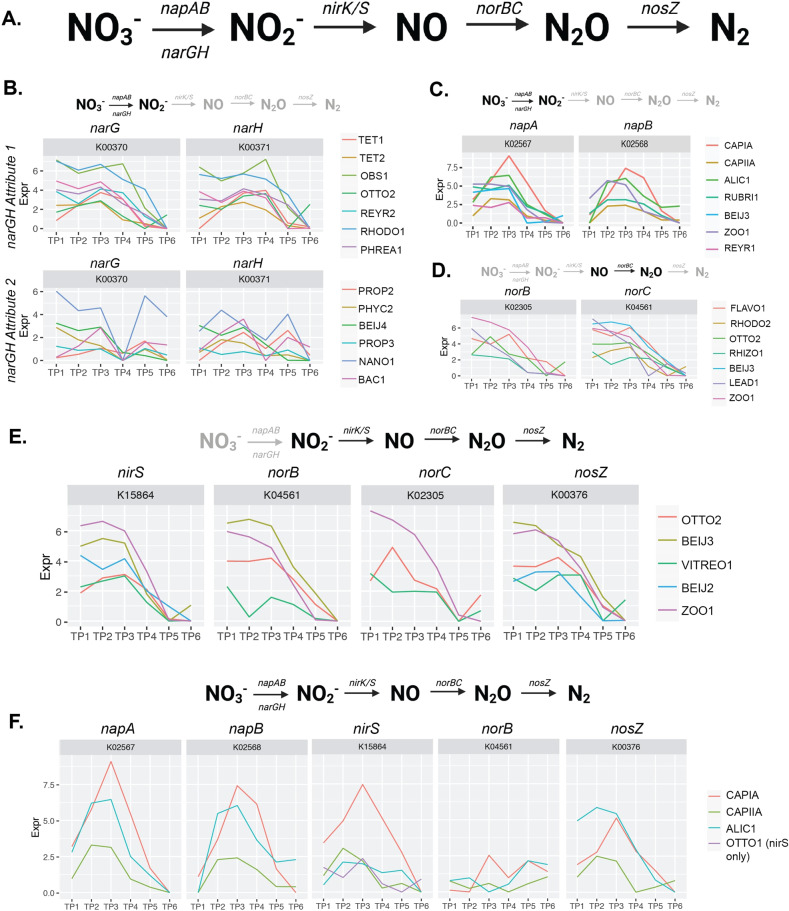
Expression dynamics of distributed denitrification routes. Expression of denitrification traits distributed among community members in the EBPR SBR ecosystem. Timepoints 1–3 correspond to the anaerobic phase and timepoints 4–6 correspond to the aerobic phase as referenced in Fig. 1. **A** Complete denitrification pathway and associated genetic repertoire with each sequential step. **B** Trait attributes of expression dynamics for community members with the *narGH* nitrate reductase system. This trait was the only denitrification trait identified with more than one attribute. **C** Expression dynamics of the *napAB* nitrate reductase system. **D** Expression dynamics of the *norBC* nitrous oxide reductase system. **E** Expression of all steps of denitrification starting at nitrite reduction. **F** Expression of the most complete denitrification route among three community members, with the *norC* subunit for nitrous oxide reduction missing. Note that OTTO1 only contains *nirS* but is included in this trait attribute because the expression dynamics are similar to that of the other three genomes for this subunit.

Denitrification gene induction is an important ecosystem property linked to the redox status of an environment. In EBPR communities, we find many genomes with diverse and incomplete denitrification pathways, distributed across many lineages denitrification steps expected in denitrifying systems (Fig. 5). Among all 66 MAGs, we did not identify any single MAG with a complete denitrification pathway consisting of the genetic repertoire necessary to fully reduce nitrate to nitrogen gas (Supplementary Fig. 5). Instead, we identified multiple groups of organisms with truncated denitrification pathways, with steps distributed among cohorts of community members (Fig. 5).

For the first steps of reducing nitrate to nitrite, we examined expression attributes of the *napAB* and *narGH* pathways (Fig. 5B, C). For the *narGH* pathway, two attributes were identified (Fig. 5B). The first *narGH* attribute was characterized by high expression in the anaerobic phase, with decreasing transcript levels by the second time point of the anaerobic phase. Genomes containing this attribute included the experimentally verified and putative PAOs *Tetrasphaera* (TET1 and TET2) and *Ca*. Obscuribacter (OBS1), respectively. The second attribute was exhibited among members of the *Actinobacteria* (PROP2, PHYC2, PROP3, and NANO1), *Proteobacteria* (BEIJ4), and *Bacteroidetes* (BAC1). The attribute identified for *napAB* was also more highly expressed anaerobically and included CAPIA, CAPIIA, ALIC1, REYR2, RUBRI1, and BEIJ3. Interestingly, this *napAB* attribute had expression patterns that quickly decreased in the first aerobic time point, suggesting a tighter regulation than Attribute 1 for *narGH*. Together, this suggests that the regulation of denitrification within the EBPR ecosystem is a niche-differentiating feature whereby the induction of denitrification pathways occurs either anaerobically or only after anaerobic carbon contact.

A smaller cohort contained the genetic repertoire to reduce nitrite to nitrogen gas and exhibited hallmark anaerobic-aerobic expression patterns (Fig. 5E) These members within the *Proteobacteria* (OTTO2, BEIJ3, VITREO1, and ZOO1) contained the *nirS* nitrite reductase, the *norBC* nitric oxide reductase, and *nosZ*, and showed highest expression of these subunits towards the beginning of the anaerobic cycle, slowly decreasing over the aerobic period to their lowest in the end of the aerobic cycle. Although BEIJ2 was lacking the *norBC* system, it contained the *nirS* nitrite reductase and *nosZ* subunit, and exhibited similar expression patterns to others in this cohort. Other *Proteobacteria* lineages only contained the *norBC* subunits but were expressed in similar fashions (RHODO2, FLAVO1, RHIZO1, and LEAD1) (Fig. 5D). Accumulibacter clades IA and IIA as well as ALIC1 were the only lineages with near-complete denitrification pathways. These lineages contained the *napAB* nitrate reductase system as mentioned above, the *nirS* nitrite reductase, *norB* (missing a confident hit for the *norC* subunit), and *nosZ*. These three lineages also exhibited hallmark upregulation of all steps in the anaerobic phase, with decreased activity after aerobic contact (Fig. 5F).

Interestingly, Accumulibacter clade IA exhibited a higher level of transcripts associated denitrification steps when expression levels were normalized relative to clade IIA, supporting the hypothesis that denitrification is a niche-differentiating feature among clades [28, 31, 74], and possibly a strain-specific trait since denitrification traits cannot be predicted based on *ppk1* clade designations [32]. For example, independent observations in differences among denitrification activities among strains within Accumulibacter clade IC are inconsistent [34, 75]. Within the same bioreactor environment, coexisting Accumulibacter clades differ between denitrification abilities and expression profiles [31–33]. Truncated denitrification pathways have also been previously shown to be distributed among community members, with the complete denitrification genetic repertoire only present in few members [32, 33], which could be due to extensive horizontal gene transfer of genes comprising denitrification steps [32, 76]. Although this experiment was not conducted under denitrifying conditions, our approach could be applied to denitrifying EBPR systems to further understand the distribution of denitrification traits among community members and how to selectively enrich for diverse DPAOs.

### Biosynthetic potential and expression dynamics of amino acid and vitamin synthesis pathways

**Fig. 6:**
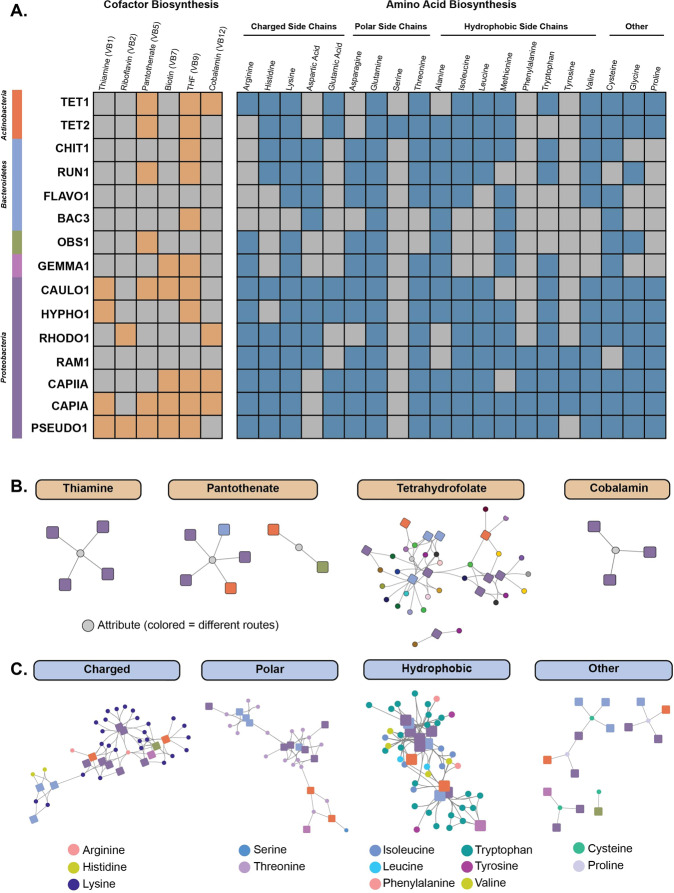
Biosynthetic potential compared to expression of amino acid and vitamin synthesis pathways for top 15 expressed MAGs. Biosynthetic potential and expression patterns of amino acid and vitamin pathways were analyzed for the top 15 genomes with the highest transcriptional counts (Table 1). **A** For a pathway to be considered present for downstream analysis in the TbasCO pipeline, 80% of the pathway had to be present in a genome. Thus, we used this cutoff criterion to discern whether a specific pathway was present or absent in a genome (with the expectation of methionine, as all genomes did not contain at least 80% of the subunits in the KEGG methionine synthase pathway, we inferred the presence of the methionine synthase as presence of this pathway). Orange colored boxes for cofactor biosynthesis pathways represents the presence of that pathway, whereas grey infers absence. For amino acid biosynthetic pathways, amino acids are listed by their side chain groups – charged, polar, hydrophobic, and other. Blue colored boxes for amino acid biosynthesis pathways represents the presence of that pathway, whereas grey infers absence. **B** Mini-networks of vitamin co-factors. Squares are genomes with the colors matching the color bar in **A**. Nodes are attributes, where the colored nodes for the tetrahydrofolate attributes represent the different routes. **C** Mini-networks of amino acid biosynthesis pathways split by type. Colors of nodes for each amino acid represent the different routes for that pathway. Squares represent genomes with colors matching the color bar in **A**.

Although SBRs are designed to enrich for Accumulibacter by providing acetate as the sole carbon source, a diverse bacterial community persists in these setups [27, 32]. One hypothesis for the persistence of these bacterial community members may be cooperative interactions due to underlying auxotrophies of amino acid and vitamin biosynthetic pathways in Accumulibacter. Amino acids and vitamin cofactors are metabolically expensive to synthesize, and widespread auxotrophies have been widely documented among microbial communities [77, 78]. Specifically, auxotrophies of vitamin cofactors have been shown to fuel bacterial and cross-kingdom interactions with *de novo* synthesizers [79, 80]. To explore this hypothesis in the EPBR SBR community, we analyzed the presence of amino acid and vitamin biosynthetic pathways and their expression patterns among the top 15 genomes based on transcript abundance (Fig. 6).

Within Accumulibacter, there are a few key vitamin cofactor and amino acid auxotrophies that could fuel potential interactions with other community members. Both Accumulibacter clade genomes are missing the riboflavin pathway for FAD cofactor synthesis, as well as known pathways for serine and aspartic acid (Fig. 6A). The biosynthetic pathway for aspartic acid is distributed among members of the *Bacteroidetes* and *Proteobacteria*, whereas only TET2 contains the pathway for serine synthesis (Fig. 5A). The lack of serine biosynthesis pathways in Accumulibacter and other genomes seems striking given that serine is one of the least metabolically costly amino acids to synthesize [81]. Interestingly, Accumulibacter clade IIA (strain CAPIIA) does not contain the biosynthetic machinery for thiamine and pantothenate synthesis, whereas clade IA (strain CAPIA) does (Fig. 6A). Only the CAULO1, HYPHO1, and PSEUDO1 genomes within the *Proteobacteria* can synthesize thiamine, whereas several other members can synthesize pantothenate (Fig. 6A). The absence of the pantothenate biosynthetic pathway in Accumulibacter CAP IIA is particularly interesting given that coenzyme A is essential for polyhydroxyalkanote biosynthesis, which fuels the rapid and extensive polymer cycling PAO phenotype of Accumulibacter [24].

In addition to other community members potentially supporting the growth of Accumulibacter due to underlying auxotrophies, the reciprocal logic may be possible as well. Both Accumulibacter clades contain the pathways for synthesizing tyrosine and phenylalanine, which are missing in a majority of the top 15 active non-Accumulibacter bacterial genomes (Fig. 6A). Only two other members within the *Proteobacteria* can synthesize tyrosine and phenylalanine, where RAM1 can synthesize both and PSEUDO1 only phenylalanine. Interestingly, phenylalanine and tyrosine are the second and third most metabolically expensive amino acids to synthesize, respectively, with tryptophan being the most costly [81]. Additionally, a few highly active non-Accumulibacter bacterial community members lack the biosynthetic machinery for several vitamin cofactors and amino acids, such as FLAVO1 and BAC3 within the *Bacteroidetes* and the putative PAO *Ca*. Obscuribacter phosphatis OBS1 (Fig. 6A). Particularly, RAM1 within the *Proteobacteria* is missing the biosynthetic machinery for all vitamin cofactors but can synthesize most amino acids including the most metabolically expensive as mentioned above.

We next analyzed the distribution of trait-attributes of vitamin and amino acid pathways among these genomes to understand how these biosynthetic pathways are expressed similarly or differently in the EBPR SBR ecosystem (Fig. 6B, C). Members of the *Proteobacteria* containing thiamine and cobalamin biosynthetic pathways all express these traits similarly (Fig. 6B). However, the pantothenate synthesis pathway contains two trait-attributes and is expressed differently among two cohorts. In the first attribute, RUN1, TET1, CAULO1, CAPIA, and PSEUDO1 express the pantothenate pathway similarly. However, OBS1 and TET2 express the pantothenate pathway differently (Fig. 6B). Because tetrahydrofolate can be synthesized through different metabolic routes, we analyzed the differences in trait attribute expression for all routes in genomes that contained sufficient coverage of this trait. *Bacteroidetes* and *Proteobacteria* members mostly cluster together among tetrahydrofolate attributes, whereas the TET1 and TET2 genomes are differentiated (Fig. 6B).

Expression of various groups of amino acids show more differentiated expression patterns for genomes with these pathways. Several amino acids also contain different metabolic routes for biosynthesis, and we analyzed all trait attributes for each amino acid for all routes grouped by type (Fig. 6C). For the charged amino acids arginine, histidine, and lysine, *Proteobacteria* and *Bacteroidetes* members cluster within their phylogenetic groups, respectively, with lysine and histidine expressed differently among these groups (Fig. 6C). In contrast, arginine is expressed similarly among all *Proteobacteria* genomes. Among the polar charged amino acids, TET2 is the only genome among the top 15 genomes that contains the pathway to synthesize serine (Fig. 6A). Several groups contain the pathway for threonine synthesis, and expression of different threonine routes are differentiated among the *Proteobacteria, Bacteroidetes*, and *Tetrasphaera spp*., though they mostly cluster phylogenetically (Fig. 6C). Notably, the expression patterns for the cysteine and proline biosynthetic pathways do not cluster phylogenetically, such as both *Tetrasphaera* genomes expressing the proline pathway more similarly to other *Proteobacteria* and *Bacteroidetes* (Fig. 6C). The few lineages that can synthesize tyrosine and phenylalanine (CAPIA, CAPIIA, RAM1, PSEUDO1) show different expression patterns. These results show that beyond the presence or absence of key vitamin cofactor and amino acid biosynthetic pathways, EBPR SBR organisms also display coherent and differentiated expression patterns for these traits, of which the functional consequences remain to be further understood.

## Conclusions and future perspectives

In this work, we applied a novel trait-based ‘omics pipeline to a semi-complex, engineered bioreactor microbial community to explore ecosystem-level and niche-differentiating traits. Through recovering 66 MAGs from the EBPR SBR community and using a time-series metatranscriptomics experiment, we were able to extend functional predictions such as identifying multiple attributes of high-affinity phosphate transporters beyond hypotheses made from traits alone. We extended this framework to other significant traits that are distributed among community members such as denitrification and amino acid metabolism. Specifically, we demonstrate that traits with similar expression profiles may be clustered into attributes providing a new layer to trait-based approaches.

We believe that identifying expression-based attributes will be a powerful tool to explore microbial traits in natural, engineered, and host-associated microbiomes. Outside of activated sludge systems, trait-based approaches could illuminate how similar secondary metabolite clusters are expressed among different species in a community [82, 83], how auxotrophies for amino acid and vitamin cofactors govern interactions [84], how rhizosphere microorganisms respond to day-night cycles, and identify putative traits that universally exhibit ecosystem-level or niche-differentiating patterns across ecosystems [19, 23]. Importantly, our trait-based approach can be used to screen for expected expression patterns of a key trait compared to a model organism, and then prioritize specific microbial lineages for downstream experimental verification with techniques such as Raman-FISH [85, 86].

## Supplementary information

Supplemental Material

Supplementary Figure 1

Supplementary Figure 2

Supplementary Figure 3

Supplementary Figure 4

Supplementary Figure 5

Supplementary Figure 6

Supplementary Figure 7

## Data Availability

All supplementary files including functional annotations and transcriptome count files are available at https://figshare.com/projects/EBPR_Trait-Based_Comparative_Omics/90437. All 64 genomes have been deposited in NCBI at Bioproject PRJNA714686. The remaining two reassembled Accumulibacter genomes have not been deposited in NCBI to not confuse between the original CAPIA and CAPIIA assemblies [27, 28]. These contemporary assemblies are available at the Figshare repository. The three metagenomes and six metatranscriptomes used in this study are available on the JGI/IMG at accession codes 3300026302, 3300026286, 3300009517, and 3300002341-46, respectively. All code for performing metagenomic assembly, binning, and annotation can be found at https://github.com/elizabethmcd/EBPR-MAGs. The TbasCO method has been implemented as a reproducible R package and can be accessed at https://github.com/Jorisvansteenbrugge/TbasCO.
