# Supplementary figures and images for: TbasCO: trait-based comparative ‘omics identifies ecosystem-level and niche-differentiating adaptations of an engineered microbiome
Source: ISME Commun. 2022 Nov 7;2:111. doi: 10.1038/s43705-022-00189-2 (PMC9723799; doi:10.1038/s43705-022-00189-2)

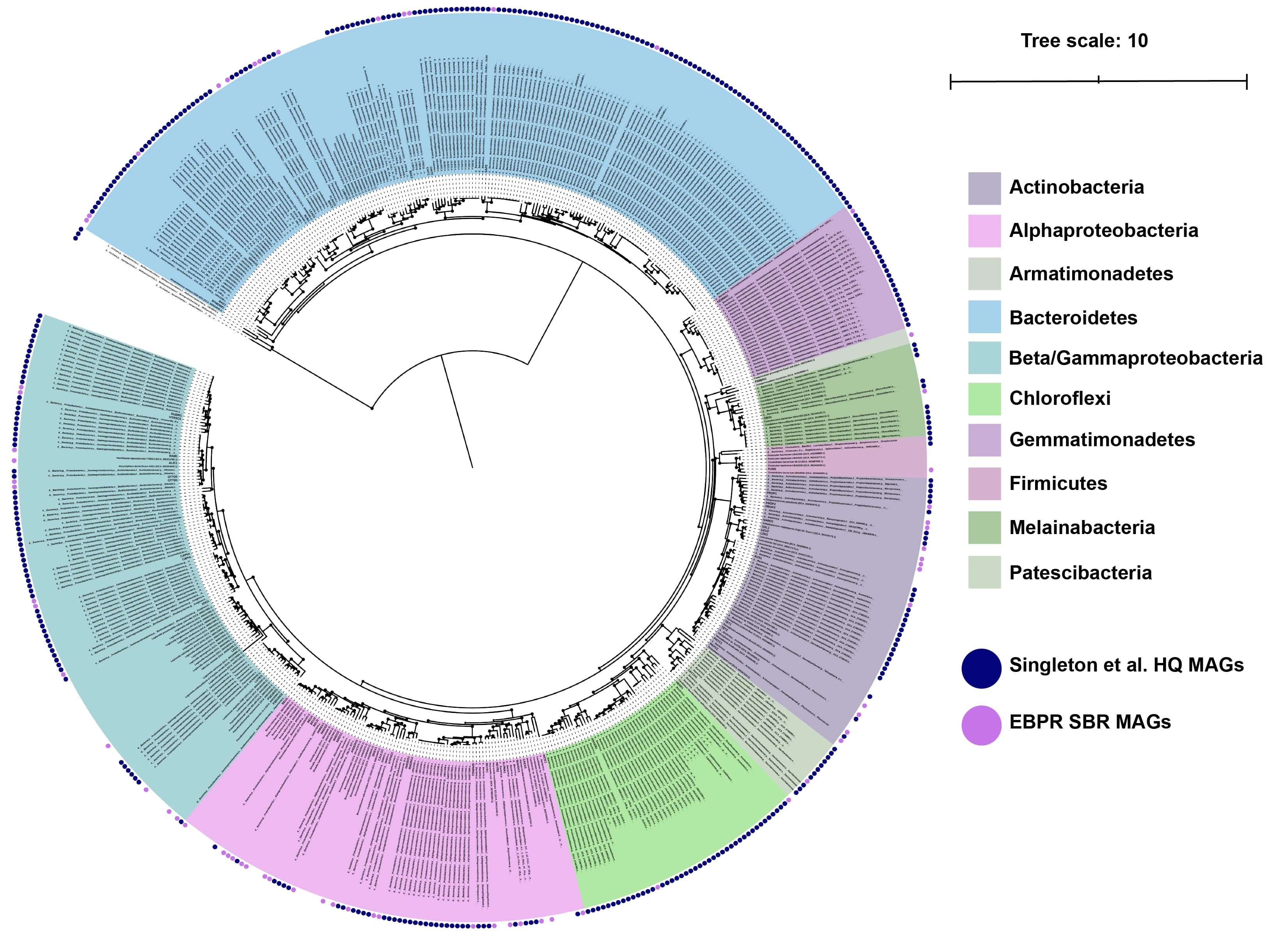

Supplement: Supplementary file 2 — Supplementary Figure 1 [file 43705_2022_189_MOESM2_ESM.jpg]

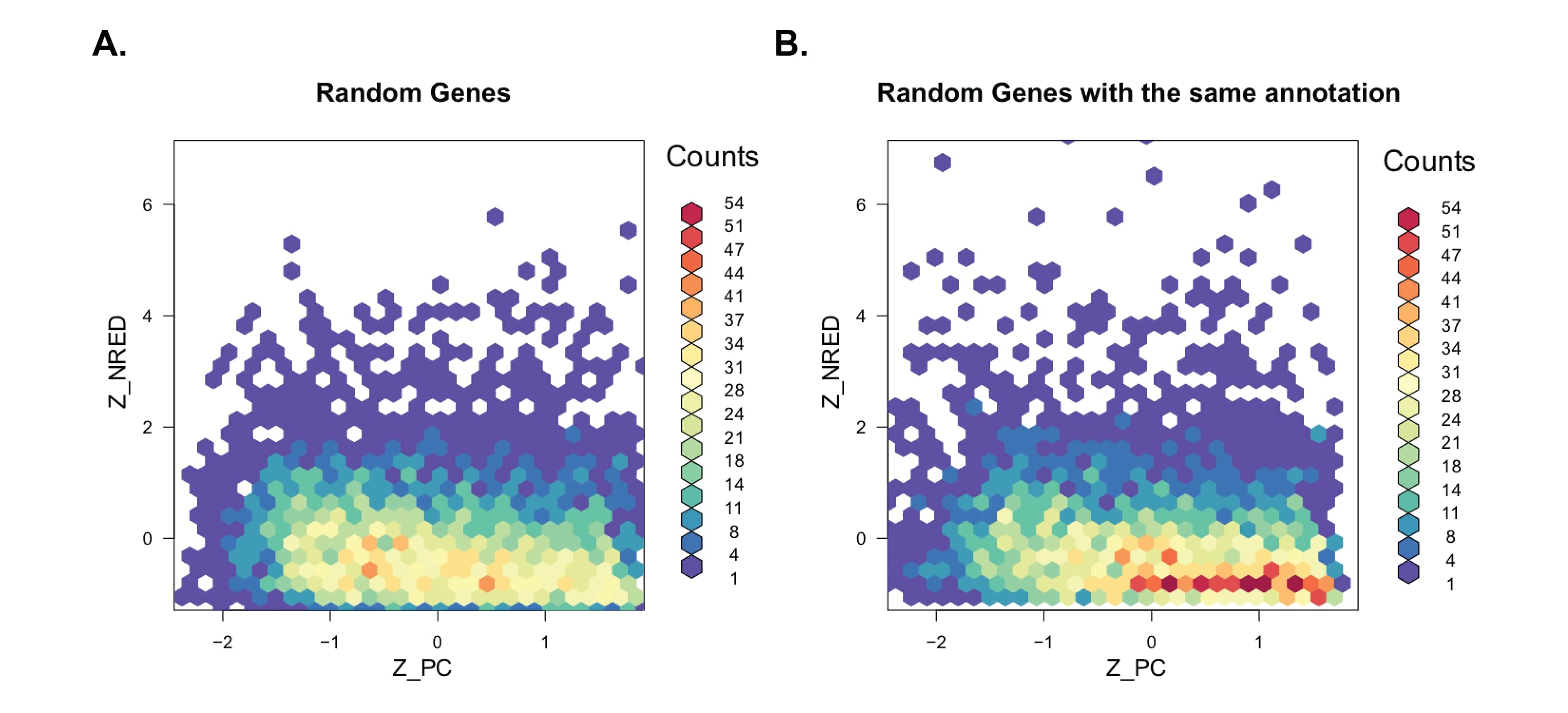

Supplement: Supplementary file 3 — Supplementary Figure 2 [file 43705_2022_189_MOESM3_ESM.jpg]

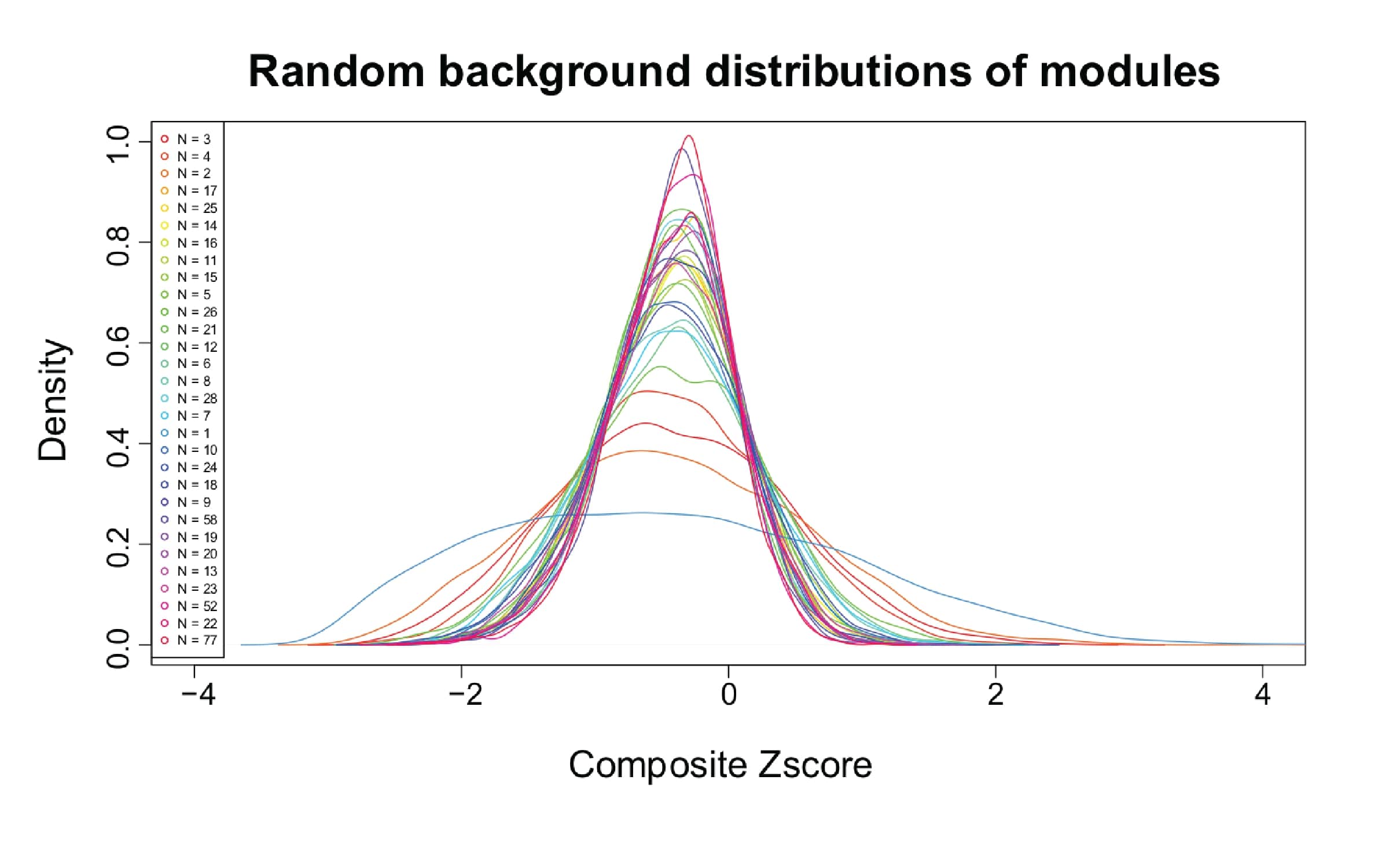

Supplement: Supplementary file 4 — Supplementary Figure 3 [file 43705_2022_189_MOESM4_ESM.jpg]

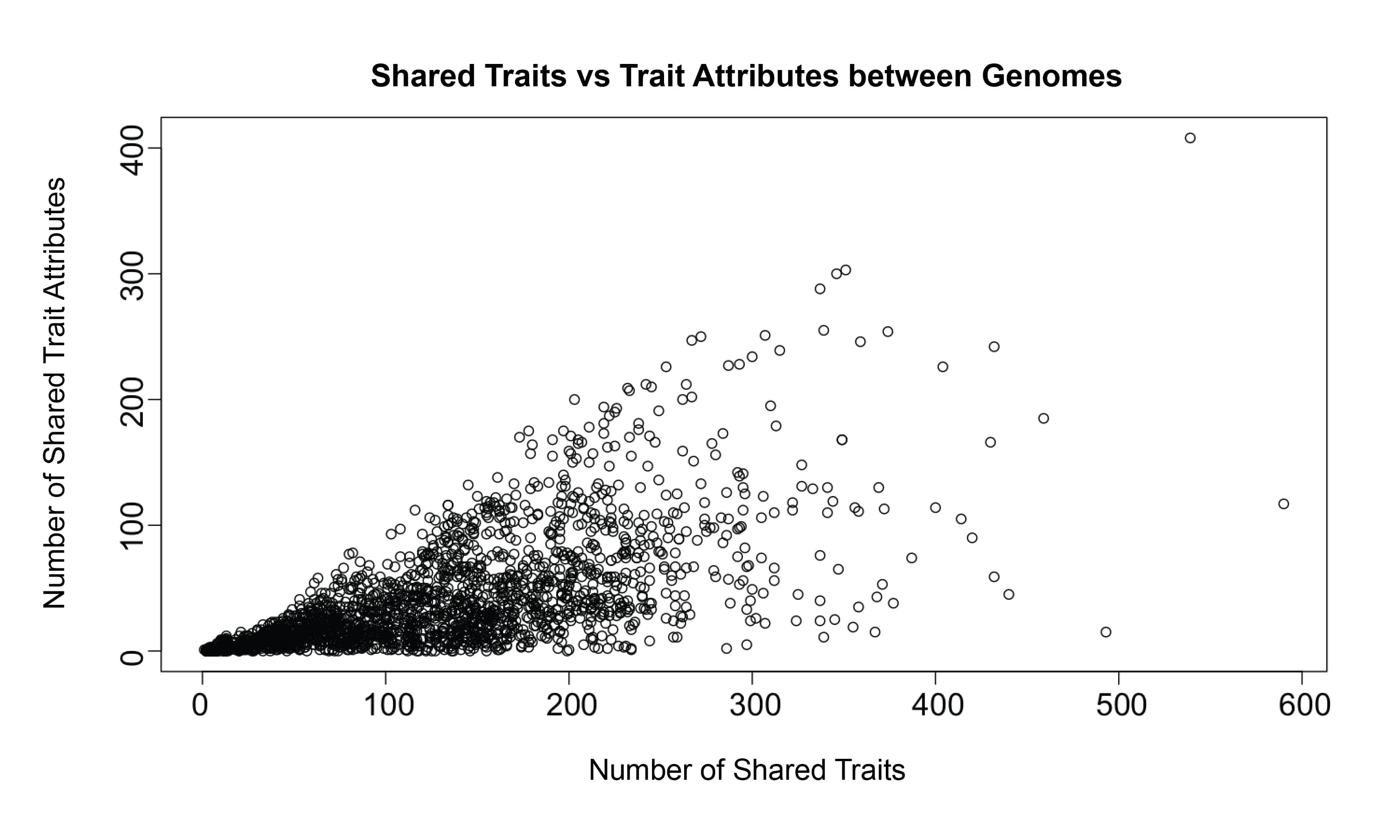

Supplement: Supplementary file 5 — Supplementary Figure 4 [file 43705_2022_189_MOESM5_ESM.jpg]

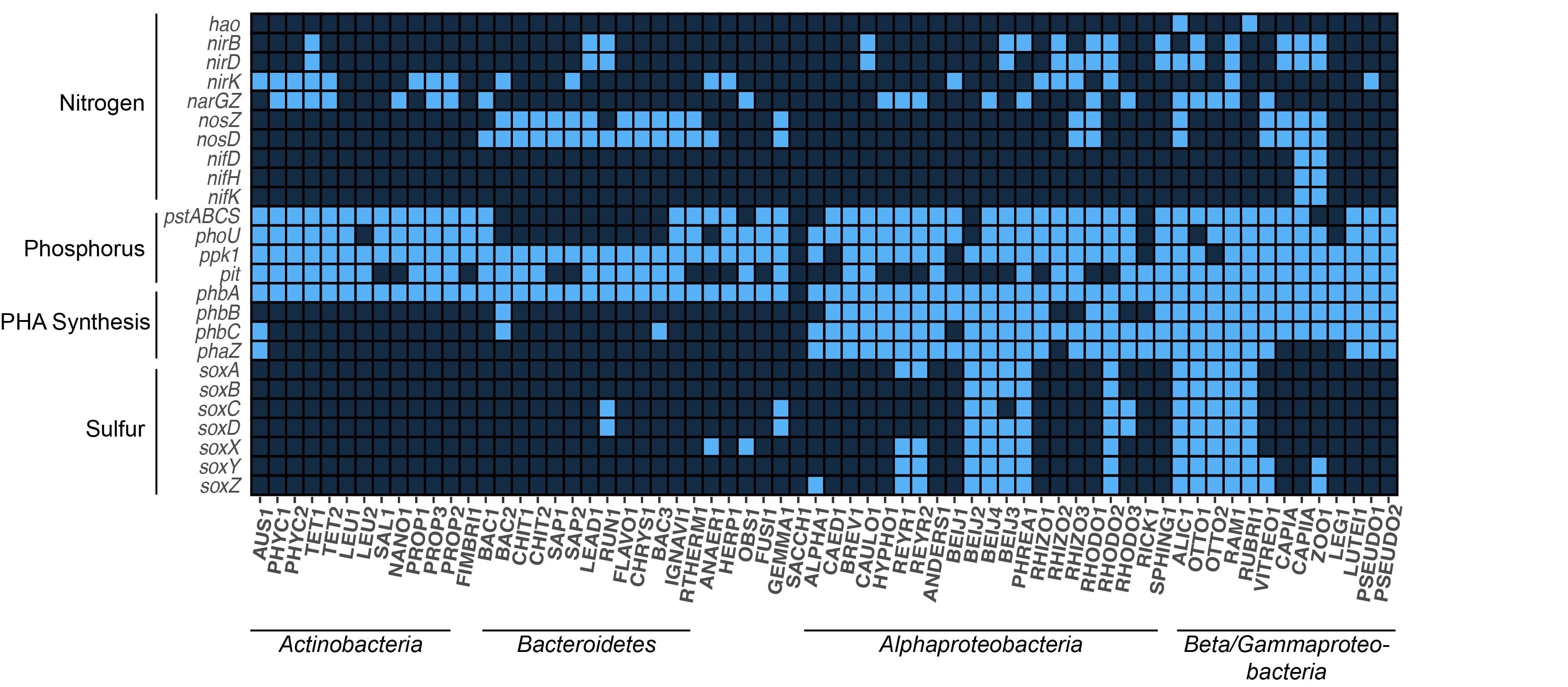

Supplement: Supplementary file 6 — Supplementary Figure 5 [file 43705_2022_189_MOESM6_ESM.jpg]

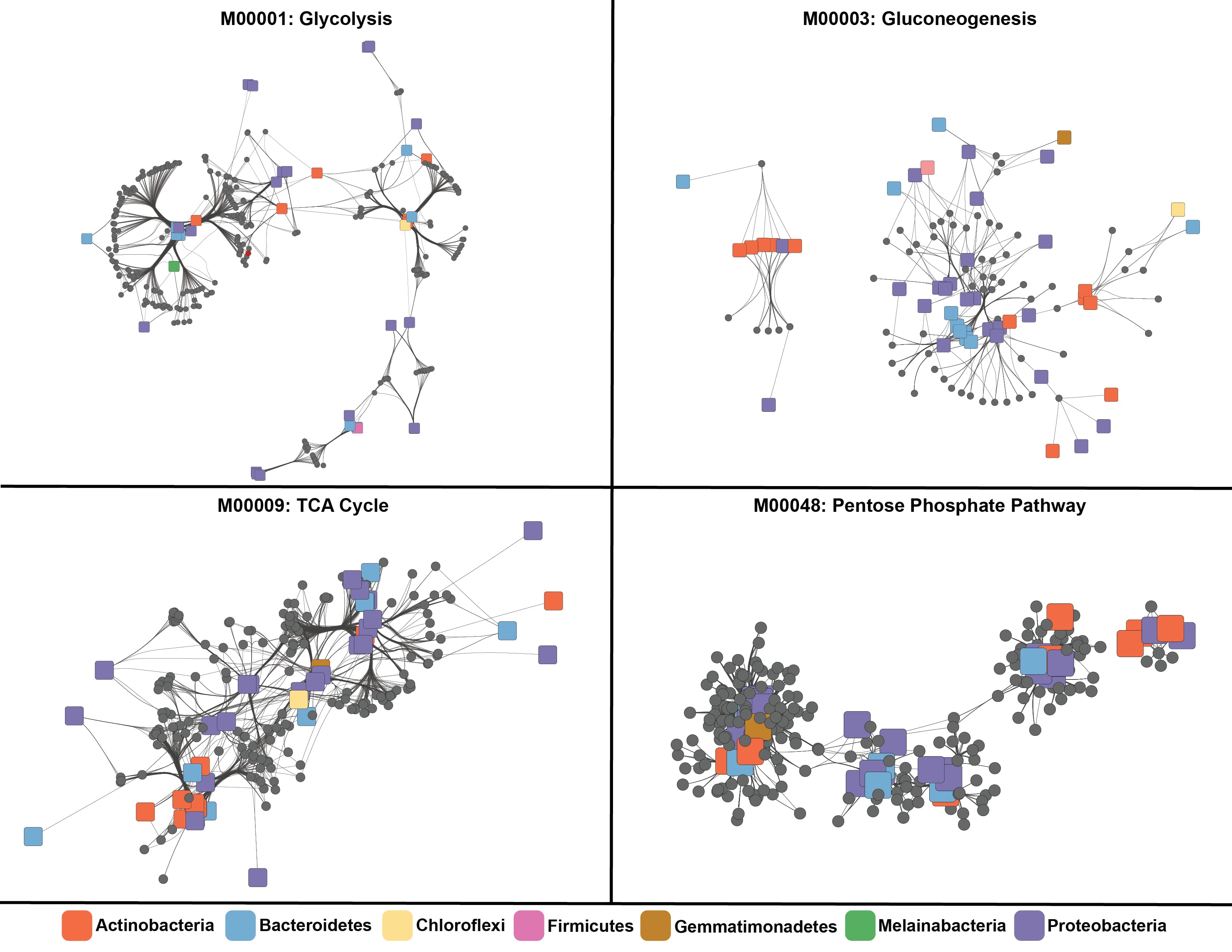

Supplement: Supplementary file 7 — Supplementary Figure 6 [file 43705_2022_189_MOESM7_ESM.jpg]

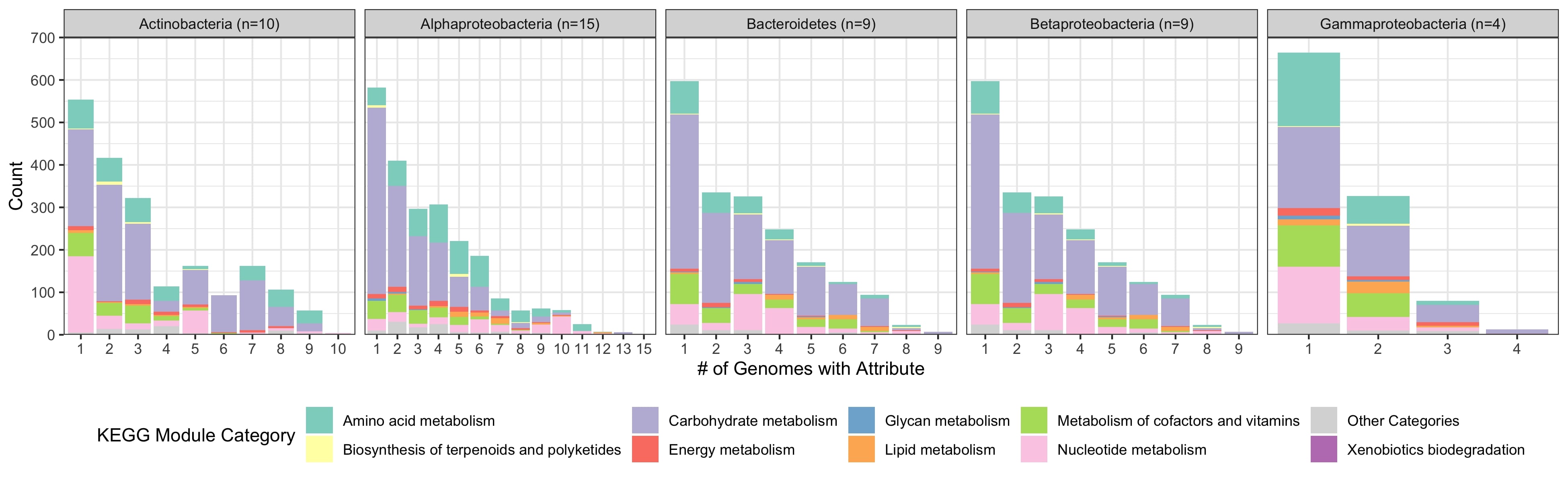

Supplement: Supplementary file 8 — Supplementary Figure 7 [file 43705_2022_189_MOESM8_ESM.jpg]
